# Beyond Recovery: Exploring Growth in the Aftermath of Psychosis

**DOI:** 10.3389/fpsyt.2020.00108

**Published:** 2020-02-26

**Authors:** Ying Ying Lee, Swapna Verma, Mythily Subramaniam

**Affiliations:** ^1^ Research Division, Institute of Mental Health, Singapore, Singapore; ^2^ Department of Psychosis, Institute of Mental Health, Singapore, Singapore; ^3^ Office of Education, Duke-National University of Singapore Medical School, Singapore, Singapore; ^4^ Neuroscience and Mental Health, Lee Kong Chian School of Medicine, Singapore, Singapore; ^5^ Saw Swee Hock School of Public Health, National University of Singapore, Singapore, Singapore

**Keywords:** psychosis, growth, post-traumatic growth, recovery, schizophrenia

## Introduction

“I'm unraveling,” I said to my psychiatrist.

“What do you mean? Tell me more,” she inquired.

“I feel … my personhood is falling apart … like a ribbon coming undone, unraveled,” I replied.

Haltingly, I added that I was treading between reality and delusions. I felt like at any moment, I was going to be swept away by the torrential outpouring of psychotic thoughts in my mind. I couldn't stop crying.

All my efforts at rebuilding my life after my first psychotic episode were coming apart. I was in pieces again. What will become of my future? I shuddered at that thought.

That was 5 years ago.

After that second episode, I completed my doctoral thesis (1), defended my research to a panel of distinguished neuroscientists, and relocated home. After a 2-year stint as a peer support specialist at a tertiary psychiatric hospital (2), I joined its research department (3). My current research efforts endeavor to understand recovery and growth from first episode psychosis. Informed by my lived experience with schizophrenia, I am convinced that growth, in the aftermath of psychosis, is possible.

That growth happens in suffering is not a new idea. Friedrich Nietzsche's view on trauma—what does not kill us makes us stronger—is a well-known maxim. Most millennials in the English-speaking world would have grown up listening to Kelly Clarkson's hit song *Stronger (What doesn't kill you)*. Any entomologist will tell you that only butterflies that have struggled out of their cocoons can fly. Traumatic experiences, uncomfortable as they are, play a central role in subsequent growth and development of a living creature. Post-traumatic growth (PTG) is the scientific study of this curious phenomenon in humans. Coined by Tedeschi and Calhoun in the 1990s, PTG is defined as “[the] positive psychological changes experienced as a result of the struggle with highly challenging life circumstances” (4).

### Post-Traumatic Growth Entails Three Levels of Positive Change

In a bid to measure the perceived positive outcomes experienced by people with traumatic experience, Tedeschi and Calhoun conducted an extensive literature review (5). They identified three levels of positive changes experienced by people with traumatic experiences over time: intrapersonal changes, interpersonal changes, and suprapersonal changes.

Intrapersonal changes stem from a deeper understanding of one's limits and strengths (personal strength). Tedeschi and Calhoun described this positive change as being “more vulnerable, yet stronger” (6). That traumatic experiences could leave a person feeling the full extent of his or her fragility is not surprising. However, the paradox is the inner strength that one uncovers in the process of overcoming the obstacles tells of the hidden strengths and resources that he or she is previously unaware of.

Interpersonal changes come from stronger bonds with family and friends (relating to others). Crisis makes or breaks relationships. In times of trouble, a person may have friends or family members who abandon them. However, there may also be people who remain supportive of them. The disaster brought on by challenging life events prunes superficial connections and strengthens genuine relationships, resulting in a milieu filled with people who truly matter.

Suprapersonal changes are outward expressions of the intrapersonal and interpersonal changes. It goes beyond one's immediate personal life. It may involve a positive change in one's outlook in life and the development of one's spirituality (appreciation for life, new possibilities, and spirituality). This may include not taking life for granted, exploring new skills and interests in life, and having a more balanced view on spirituality and faith.

Based on these domains, the Post-Traumatic Growth Inventory (PTGI) was developed and validated in an initial sample population (5). Today, this inventory is used widely by researchers from different fields, on people with different kinds of trauma: survivors of natural disasters (7, 8), sexual abuse (9), cancer (10), suicide survivors (11), and even psychosis (12, 13). The five domains of PTGI include personal strength, new possibilities, relating to others, appreciation of life, and spiritual change (5). This growth happens only when the traumatic event causes significant psychological distress and nullifies the person's initial worldwide (i.e., the person's ideas and assumptions on the world were proven to be wrong) (4, 6).

### Post-Traumatic Growth in Psychosis

Psychotic episodes are traumatic experiences. The symptoms of this illness—delusions, hallucinations, disorganized behaviors and more—can cause a lot of distress to its patients. In fact, many reported fear and anxiety during their episodes (14). A systematic review on post-traumatic experiences of persons with first episode psychosis estimated the pooled prevalence of post-traumatic stress disorder (PTSD) symptoms at 42%, and PTSD diagnosis at 30% (15). In Singapore, a study on patients recovering from first episode of psychosis estimated the prevalence of PTSD at 19.7% (16). Yet, two independent studies conducted in the UK and Canada suggested that post-traumatic distress may not be the sole outcome in the aftermath of psychotic episodes (12, 17).

The British team built a framework for PTG from the recovery narratives of persons with psychosis and other serious mental health issues (17). Six themes were generated from the semi-structured interviews with mental health patients: self-discovery, sense of self, life perspective, wellbeing, relationships, and spirituality. The authors concluded that growth was a part of the recovery narratives of persons with serious mental health issues. The Canadian team published a mixed methods study on PTG in persons with first episode psychosis (12). Their qualitative analysis showed that besides distress from psychosis, the participants also experienced positive changes such as a stronger sense of self, more balanced view of religiosity, and improvements in health and personality.

Readers familiar with Tedeschi's work would find the findings from the UK and Canada teams reminiscent of the five domains in PTGI. Even though every participant in the studies has his or her unique trauma story, the positive changes that result from their experiences fall into more or less consistent domains. The PTG framework developed by Tedeschi and colleagues in the 1990s holds true today and could be applied in the context of mental health research.

Conceptually, PTG has been contrasted with resilience by Tedeschi and colleague (4). Both PTG and resilience have some similarities, as both phenomena encompass the idea of thriving in adversity. However, they are conceptually different. Resilience has more to do with a person displaying positive adaptation *despite* significant trauma or adversity (18). PTG postulates that a person becomes psychologically stronger *because* of a significant trauma or adversity (4). Even though the outcomes of resilience and PTG (namely positive growth and adaptation) may appear to be identical, the processes in which these phenomena develop may be different. Researchers of resilience suggested that resilience is an innate characteristic (18), while PTG is a process that may be facilitated by healthcare workers (6). Hence, PTG and resilience are related, but distinct concepts.

To date, most published studies on PTG in psychosis are at the descriptive level. Even though they have laid a foundation for the presence of growth after psychosis, they have yet to explore the mechanism(s) behind its occurrence, and its relationship with related concepts such as resilience and recovery.

### Beyond Survival

The American survivor culture has shaped therapy goals for many people with traumatic experiences to move from a victim mentality toward a survivor mentality (19). While the initial identification of the person as a victim, and then as a survivor, serve their respective purposes for help-seeking and for overcoming the immediate psychological damage, Dolan (19) noticed that staying in the survivor mode was not sufficient for her clients to thrive. A lot of them live against the backdrop of a mild depressive state, even if they managed to regain some semblance of life. At the same time, she encountered some clients with a history of abuse, yet leading meaningful and fulfilling life against all odds. When she interviewed them, she noticed that they do not refer to themselves as victims, or even survivors of their past. They seemed to have created new identities—free from their past, more toward to their authentic selves. She proposed that working beyond the survivor mentality, carving out a new sense of self, was the missing key for many of her clients (19). This involves growing beyond a traumatic past toward thriving.

### Implications of Post-Traumatic Growth on Mental Healthcare

The implications of these growth findings are tremendous. It could change the way one thinks about the trajectory of serious mental illness and change how one treats these issues in the clinical setting. In the last decades, research on recovery from serious mental health issues grew exponentially (20–23). There is now a strong body of evidence that supports the notion of recovery from serious mental illness. That mental illness is not a one-way ticket into an abysmal depth of disability—but of recovery—is widely accepted by the community. However, the current understanding of the detriments brought on by mental illness, and the subsequent recovery, may be incomplete. Like Dolan's idea of victim-survivor-authentic self in trauma care, I would propose that there is more beyond recovery for persons with serious mental illness; that PTG is possible for them, because of the crisis that they went through during the condition. The seismic shift brought on by mental illness in a person's life may be viewed as a unique opportunity to re-evaluate one's strengths and weaknesses, to strengthen bonds with people who truly matter, and to connect with a purpose larger than oneself. Thus, a psychiatric condition could be a trigger for psychological growth. However, this growth does not happen by chance. To catalyze it, new dimensions have to be added to the roles of mental health professionals. Instead of limiting clinical work to rehabilitation and recovery, intentional efforts could also be made to capitalize the patients' traumatic experiences with mental illness and turn it into a growth opportunity.

The challenge remains for us to understand how growth may be facilitated after mental health conditions. Tedeschi and Calhoun proposed that cognitive rumination, coupled with appropriate self-disclosure and social support, produces a personal narrative shift in PTG (4). Janoff-Bulman (24) expounded on the importance of making sense of traumatic experiences in the development of PTG. There may be some hidden treasures waiting to be uncovered from the body of work on PTG that could be applied to mental healthcare practices.

Granted, not everyone who survives mental illness grows from the experience. It does not mean that PTG is reserved for the privileged few. In the context of mental healthcare, I believe that opportunities for growth need to be intentionally introduced into the recovery plans of persons with mental illness. To grow in the aftermath of a mental illness, a period of introspection, positive self-disclosure, and benefit finding needs to be injected into a person's life. Deeper insights into how exactly PTG develops after mental health conditions, and how mental health professionals can support their patients to achieve that, could bring existing mental healthcare services to greater heights.

Looking back, it is almost like the three psychotic episodes I have experienced were preparing me for the work that I do today. In many ways, I am expectant for the next 5 years. No longer do I shudder at the thought of my future, knowing that the psychological growth that ensued from my psychotic episodes has made me a stronger person.

## Author Contributions

YL conceptualized the manuscript and wrote the first draft. SV and MS gave intellectual inputs based on their clinical and research expertise on psychosis.

## Conflict of Interest

The authors declare that the research was conducted in the absence of any commercial or financial relationships that could be construed as a potential conflict of interest.

## References

[1] Lee YY Bone Morphogenetic Protein signaling in structural plasticity of cerebellar mossy fibers. Switzerland: University of Basel (2015). Retrieved from http://edoc.unibas.ch/diss/DissB_11278.

[2] Lee YY (2018). Commentary: Schizophrenia, a life increasingly detached from reality. CNA. Retrieved from https://www.channelnewsasia.com/news/commentary/commentary-schizophrenia-a-life-increasingly-detached-from-10383400.

[3] Lee YY Ang S Chua HC Subramaniam M Peer support in mental health: a growing movement in singapore. Ann Acad Med Singapore (2019) 48(3):95–7. 30997478

[4] Tedeschi RG Calhoun LG Posttraumatic growth: conceptual foundations and empirical evidence. Psychol Inq (2004) 15(1):1–18. 10.1207/s15327965pli1501_01

[5] Tedeschi RG Calhoun LG The posttraumatic growth inventory: measuring the positive legacy of trauma. J Trauma Stress (1996) 9(3):455–71. 10.1002/jts.2490090305 8827649

[6] Calhoun LG Tedeschi RG Facilitating Post-Traumatic Growth: A Clinician"s Guide. US: Lawrence Erlbaum Associates Publishers (1999).

[7] Chen J Zhou X Zeng M Wu X Post-traumatic stress symptoms and post-traumatic growth: evidence from a longitudinal study following an earthquake disaster. PloS One (2015) 10(6):1–10. 10.1371/journal.pone.0127241 PMC4457873 26046912

[8] Nakagawa S Sugiura M Sekiguchi A Kotozaki Y Miyauchi CM Hanawa S Effects of post-traumatic growth on the dorsolateral prefrontal cortex after a disaster. Sci Rep (2016) 6(34364):1–9. 10.1038/srep34364 27670443 PMC5037468

[9] Kaye-Tzadok A Davidson-Arad B Posttraumatic growth among women survivors of childhood sexual abuse: Its relation to cognitive strategies, posttraumatic symptoms, and resilience. Psychol Trauma: Theory Res Practice Policy (2016) 8(5):550–8. 10.1037/tra0000103 27018919

[10] Cormio C Muzzatti B Romito F Mattioli V Annunziata MA Posttraumatic growth and cancer: a study 5 years after treatment end. Support Care In Cancer (2017) 25(4):1087–96. 10.1007/s00520-016-3496-4 PMC5321703 28013416

[11] Drapeau CW Lockman JD Moore MM Cerel J Predictors of posttraumatic growth in adults bereaved by suicide. Crisis (2019) 40(3):196–202. 10.1027/0227-5910/a000556 30375239

[12] Jordan G Malla A Iyer SN “It's Brought Me a Lot Closer to Who I Am”: a mixed methods study of posttraumatic growth and positive change following a first episode of psychosis. Front In Psychiatry (2019) 10(480):1–13. 10.3389/fpsyt.2019.00480 PMC6643164 31379615

[13] Mazor Y Gelkopf M Mueser KT Roe D posttraumatic growth in psychosis. Front In Psychiatry (2016) 7(202):1–11. 10.3389/fpsyt.2016.00202 PMC5165025 28066275

[14] Roe D Lachman M The subjective experience of peoplewith severe mental illness: a potentially crucial piece of the puzzle. Isr J Psychiatry Relat Sci (2005) 42(4):223–30. 16618053

[15] Rodrigues R Anderson KK The traumatic experience of first-episode psychosis: a systematic review and meta-analysis. Schizophr Res (2017) 189:27–36. 10.1016/j.schres.2017.01.045 28214175

[16] Sin G-L Abdin E Lee J Poon L-Y Verma S Chong S Prevalence of post-truamatic stress disorder in first-episode psychosis. Early Interv In Psychiatry (2010) 4(4):299–304. 10.1111/j.1751-7893.2010.00199.x 20977686

[17] Slade M Rennick-Egglestone S Blackie L Llewellyn-Beardsley J Franklin D Hui A Post-traumatic growth in mental health recovery: qualitative study of narratives. BMJ Open (2019) 9(6):1–10. 10.1136/bmjopen-2019-029342 PMC6609070 31256037

[18] Luthar SS Resilience in Development: A Synthesis of Research across Five Decades. In: Cicchetti D Cohen DJ, editors. Developmental Psychopathology. (2015). New York, United States p. 739–95. 10.1002/9780470939406.ch20

[19] Dolan Y Beyond Survival: Living Well is the Best Revenge (2 ed.). US: Gardner Books (2000).

[20] Davidson L The recovery movement: implications for mental health care and enabling people to participate fully in life. Health Affairs (2016) 35(6):1091–7. 10.1377/hlthaff.2016.0153 27269027

[21] Frese F Walker Davis W The consumer-survivor movement, recovery, and consumer professionals. Prof Psychol: Res Pract (1997) 28(3):243–5. 10.1037/0735-7028.28.3.243

[22] Harding C Brooks G Ashikaga T Strauss J Breier A The Vermont longitudinal study of persons with severe mental illness, II: Long-term outcome of subjects who retrospectively met DSM-III criteria for schizophrenia. Am J Psychiatry (1987) 144(6):727–35. 10.1176/ajp.144.6.727 3591992

[23] Harrison G Hopper K Craig T Laska E Siegel C Wanderling J Recovery from psychotic illness: a 15- and 25-year international follow-up study. Br J Psychiatry (2001) 178(6):506–17. 10.1192/bjp.178.6.506 11388966

[24] Janoff-Bulman R Posttraumatic growth: three explanatory models. Psychol Inq (2004) 15(1):30–4.

